# The global burden of headache in children and adolescents – developing a questionnaire and methodology for a global study

**DOI:** 10.1186/1129-2377-15-86

**Published:** 2014-12-11

**Authors:** Çiçek Wöber-Bingöl, Christian Wöber, Derya Uluduz, Uğur Uygunoğlu, Tuna Stefan Aslan, Martin Kernmayer, Heidi-Elisabeth Zesch, Nancy TA Gerges, Gudrun Wagner, Aksel Siva, Timothy J Steiner

**Affiliations:** 1 Department of Child and Adolescent Psychiatry, Medical University of Vienna, Währinger Gürtel 18-20, 1090 Vienna, Austria; 2 Department of Neurology, Medical University of Vienna, Vienna, Austria; 3 Neurology Department, Cerrahpaşa Medical Faculty, Istanbul, Turkey; 4 Department of Neuroscience, Norwegian University of Science and Technology, Trondheim, Norway; 5 Division of Brain Sciences, Imperial College London, London, UK

**Keywords:** Migraine, Tension-type headache, Burden of headache, Quality of life, Global campaign against headache

## Abstract

**Background:**

Burden of headache has been assessed in adults in countries worldwide, and is high, but data for children and adolescents are sparse. The objectives of this study were o develop a questionnaire and methodology for the global estimation of burden of headache in children and adolescents, to test these in use and to present preliminary data.

**Methods:**

We designed structured questionnaires for mediated-group self-administration in schools by children aged 6-11 years and adolescents aged 12-17 years. In two pilot studies, we offered the questionnaires to pupils in Vienna and Istanbul. We performed face-to-face interviews in a randomly selected subsample of 199 pupils to validate the headache diagnostic questions.

**Results:**

Data were collected from 1,202 pupils (mean 13.9 ± 2.4 years; 621 female, 581 male). The participation rate was 81.1% in Istanbul, 67.2% in Vienna. The questionnaire proved acceptable: ≤5% of participants disagreed partially or totally with its length, comprehensibility or simplicity. The sensitivity, specificity, positive and negative predictive values ranged between 0.71 and 0.76 for migraine and between 0.61 and 0.85 for tension-type headache (TTH). Cronbach’s alpha was 0.83. The 1-year prevalence of headache was 89.3%, of migraine 39.3% and of TTH 37.9%. The prevalence of headache on ≥15 days/month was 4.5%. One fifth (20.7%) of pupils with headache lost ≥1 day of school during the preceding 4 weeks and nearly half (48.8%) reported ≥1 day when they could not do activities they had wanted to. The vast majority of pupils with headache experienced difficulties in coping with headache and in concentrating during headache. Quality of life was poorer in pupils with headache than in those without.

**Conclusion:**

These pilot studies demonstrate the usefulness of the questionnaires and feasibility of the methodology for assessing the global burden of headache in children and adolescents, and predict substantial impact of headache in these age groups.

## Background

According to the Global Burden of Disease study 2010 (GBD2010), tension-type headache (TTH) and migraine, two primary headache disorders, are the second and third most prevalent disorders in the world and migraine is the 7th highest specific cause of adult disability worldwide [1,2]. Other headache disorders, including medication-overuse headache (MOH), may, collectively, impose similar levels of disability in adults [3,4].

The burden of headache in children and adolescents is not so well measured. In 2009, Kernick and Campbell [5] reviewed 33 studies on the impact of headache in children. They found a wide range of methodologies, settings and outcome measures, and described considerable methodological limitations, but concluded that "the impact of headache in children and adolescents was substantial". They argued: "Rigorous studies are required to quantify this burden using measures that are valid and reliable and whose development has been informed by both theoretical and practical perspectives". A review published four years later identified 64 cross-sectional studies of the prevalence of headache and/or migraine in children and adolescents [6], performed in 32 countries and including a total of 227,249 participants. The estimated overall mean prevalence of headache was 54.4% (95% CI: 43.1-65.8) and of migraine 9.1% (95% CI: 7.1-11.1). Yet information about the burden of headache in these studies was limited, and there was a lack of population-based studies from low- and lower-middle income countries.

We plan a global study of the burden of headache in children aged 6-11 years and adolescents aged 12-17 years to fill this major knowledge gap. Meanwhile, guidelines for population-based studies of the burden of headache [7] have been developed by the Global Campaign against Headache [8], together with an instrument, the headache-attributed restriction, disability, social handicap and impaired participation (HARDSHIP) questionnaire, which has been used and validated in adult studies in multiple countries, languages and cultures [9]. We have built on these, in collaboration with the Global Campaign, to develop questionnaires and a survey methodology specifically for these age groups.

The basic study design is a cross-sectional survey conducted by self-completed questionnaire administered in schools selected by a partly purposive and partly convenience-based multinational modified cluster sampling technique. The participating countries will be drawn from all regions of the world. Schools will be selected by the local investigators in each country, to reflect geographical, cultural and (where relevant) ethnic diversities. Accordingly, this global study will provide fundamental information about headache disorders in children and adolescents worldwide, and it will embrace low- and lower-middle income countries which have so far been neglected.

The purpose of this paper is to describe the development of the Child and Adolescent HARDSHIP questionnaires, our assessment of their feasibility in use in schools in the manner proposed, the validation of the diagnostic questions, and our preliminary findings on the burden of headache and quality of life obtained in two pilot studies in two countries.

## Methods

The study was approved by the Ethics Committee of the Medical University of Vienna, Austria, on September 27^th^, 2012 and by the Ethics in Research Committee of the Education Ministry, Ethical Committee Istanbul, Turkey on October 17^th^, 2012. In Vienna, written informed consent was required by the ethics committee not only from all participants but also from their parents, whereas in Istanbul consent was necessary from participants and the school authorities.

### Questionnaire

In developing the questionnaires, we identified four essential requirements. First, each must be fully comprehensible to the age group for whom it was intended; second; each must collect comprehensive information; third, the findings must be reliable; and fourth, the time in the class needed by the mediator to explain the questionnaire and by the participants to fill it in should be limited (we set a maximum of 45 minutes).

We developed the Child and Adolescent HARDSHIP questionnaires as similar structured questionnaires for mediated-group self-administration by pupils in school. We based them on the original HARDSHIP questionnaire [9], to include (a) demographic enquiry, (b) questions on headache prevalence, (c) questions required for diagnosing migraine, TTH and MOH according to ICHD-II criteria [10] and (d) questions on burden attributable to headache and (e) quality of life. Only for the pilot study, we added further questions to assess the feasibility of the questionnaire.

We kept demographic questions to a minimum, asking only about age and gender. We agreed that the reliability of children’s and adolescents’ responses about their social and economic background might be limited, and the time would be better spent on other enquiries.

To screen for headache, we asked about its lifetime and 1-year prevalence. To diagnose headache type according to ICHD-II criteria [10] required questions about headache frequency and duration, headache characteristics, associated symptoms and use of acute medication.

In order to assess the impact of headache in children and adolescents, we began by collecting and reviewing available questionnaires that might suit this purpose. Among these, the paediatric version of the migraine disability assessment questionnaire (PedMIDAS) [11] had been found appropriate for epidemiological studies [12], although it was recommended to use other instruments for headache-related distress and quality of life [12]. Recently, two studies showed important limitations of PedMIDAS: first, that it underestimated the impact of migraine on non-school days [13] and, second, that the recall accuracy of PedMIDAS compared to a headache diary was better at 30 days than at 90 days [14]. For our questionnaire, we adopted elements from PedMIDAS [11] and took additional questions from KINDL® [15]. The latter is a series of questionnaires on health-related quality of life and the impact of various diseases such as asthma, epilepsy and diabetes mellitus in children and adolescents [16]; we selected those questions from it that seemed most appropriate for assessing the burden of headache (there is no specific KINDL® questionnaire for headache or migraine) and also the questions on quality of life. Burden questions adopted from PedMIDAS required responses in numbers of days. All questions taken from KINDL® were rated on a 4-point Likert scale (never, sometimes, often, always) and referred to the preceding 4 weeks.

To assess the feasibility of the questionnaire, we added questions to record starting and finishing times, and the participants were asked to rate the length and comprehensibility of the questionnaire as well as their ability to answer the questions easily, using 4-point smiley-face scales.

The questionnaires were prepared in English and translated into German and Turkish following *Lifting The Burden*‘s translation protocol [17]. Translations of questionnaires are of crucial importance, since exact meaning rather than simple linguistic equivalence must be captured.

### School studies

We selected schools by a convenience-based sampling technique in Vienna and Istanbul. Within each class in each school, all children were included, except those who refused or were unable to take part for any reason or were absent from school on the day of the interview.

The Child or Adolescent HARDSHIP questionnaire was administered to the participants in class by a physician-investigator (the mediator), introduced to the class by the teacher. The mediator explained the questionnaire, read aloud each single question, gave support and took steps to prevent participants copying responses from each other.

### Face-to-face interview

To assess the diagnostic validity of the questionnaire, we performed face-to face interviews in random subsamples of participants with migraine or TTH according to questionnaire diagnoses. All interviews were performed within one month (in order that there should not be change in the illness). For the first version of the questionnaire, we interviewed 50 subjects aged 11-17 years (mean 14.0 ± 1.9 years) in Vienna. For the second (and final) version of the questionnaire, we interviewed 59 pupils aged 7-11 years (mean 8.5 ± 1.2 years) in Vienna and 90 pupils aged 14-17 years (mean 15.7 ± 0.9 years) in Istanbul. All interviews were performed by experienced headache specialists (for the first version by CW, for the second version by HEZ in Vienna and by DU in Istanbul) who were blinded to the questionnaire diagnoses.

### Statistical analyses

The diagnostic accuracy of the questionnaire was assessed by calculating sensitivity, specificity, positive predictive value (PPV) and negative predictive value (NPV) for migraine and TTH using the face-to-face interview as gold standard. In addition, we calculated the kappa coefficient to analyze the overall agreement of headache diagnoses (*ie*, migraine (definite or probable), TTH (definite or probable), headache on ≥15 days/month and no headache) between questionnaires and interviews.

Internal consistency was analyzed by calculating Cronbach’s alpha using separately the data from the first and second versions of the questionnaires (Additional files 1 and 2) and including all but five questions (date, age, gender, headache quality and headache localization).

For parametric variables, we applied Student’s t-test and calculated Pearson’s correlation coefficients, and for nonparametric variables we used Kruskal-Wallis tests and Spearman’s correlation coefficients. We analyzed categorical variables by means of chi-squared tests.

We used IBM SPSS Statistics, Version 20, for all calculations, and set the level of statistical significance to p < 0.05. Because this was a pilot study and all analyses were explorative, we did not apply corrections for multiple testing.

## Results

### Participants

The data were collected between May 2012 and June 2013 from a total of 1,202 children and adolescents aged 6-17 years (mean 13.9 ± 2.4 years; 621 female, 581 male). In Istanbul, we included 718 participants aged 11-17 years (mean 14.5 ± 1.6 years; 354 female, 364 male) from 15 classes in two schools; in Vienna, there were 484 participants aged 6-17 years (mean 12.9 ± 3.0 years; 267 female, 217 male) from 24 classes in five schools. The participation rate was 81.1% in Istanbul and 67.2% in Vienna.

### Development of the child and adolescent HARDSHIP questionnaires

The initial version of the questionnaire, developed in 2012, was tested in 711 pupils aged 10-17 years (mean 13.7 ± 1.5 years). The agreement between questionnaire and interview with respect to diagnosis was less than 50% for headache on ≥15 days/month, frequent use of headache medication and quality of headache. Mean agreement for all other diagnostic questions was 78.1% (range 70.6-100%). The poor agreement with respect to frequent headache and medication use was due to overestimations by the questionnaire. We changed the wording of the headache frequency question from "How often do you have a headache?" (response options: All the time; Most days; Every week; Every month; Less than once a month) to "On how many days in the last four weeks did you have a headache? Please enter the number of days, between 0 and 28." We changed the medication-use question from "When you have a headache, do you take medicine or pills for it?" (response options: Every day; Most days; Every week; Every month; Less than once a month) to "On how many days in the last four weeks did you take medicine or pills because of headache? Please enter the number of days, between 0 and 28." We also changed the position of these questions within the questionnaire, and added questions referring to the previous week in order to allow comparison of 1-week and 4-week entries for assessing consistency of the answers. Furthermore, we added three questions about headache yesterday. Finally, we deleted three burden of headache questions ("I was left out by my classmates or friends when they did things together, because of my headaches"; "I felt ashamed because of my headaches"; "I was teased by other children because of my headaches") which were answered "never" by almost all (92.8-97.2%) of the participants.

The second (final) versions of the questionnaire were tested in 491 pupils, 122 children from Vienna, mean age 8.7 ± 1.3 years, and 369 adolescents from Istanbul, mean age 15.8 ± 0.9 years. Compared to the first version, the proportion of participants with headache on ≥15 days/month declined from 14.6% to 4.5%. In addition, the number of headache days given for the previous 4 weeks was consistent with the number given for the previous week (4.3 ± 4.8 *vs* 1.3 ± 1.5; Pearson correlation coefficient 0.81, p < 0.001). Even more pronounced, the proportion of subjects with possible MOH declined from 13.2% with the first version of the questionnaire to 0.8% with the revised version. Similarly to headache frequency, the entries for days with medication intake during the previous 4 weeks were consistent with those for the past week (1.5 ± 3.0 *vs* 0.5 ± 1.2 days; Pearson correlation coefficient 0.58, p < 0.001).

Scrutiny of all other questions revealed no need for further changes, except deleting the questions about the questionnaire. The final versions of the Child and Adolescent HARDSHIP questionnaires for use in the global study are added as Additional files 1 and 2.

In order to record details about the country and the school, we also developed a short questionnaire to be completed centrally and by the teacher or mediator (Additional file 3). To guarantee complete documentation of non-participation and, as far as possible, reasons for it (*eg*, withheld parental consent, absent on the day), we developed a further very brief questionnaire also to be completed by the teacher or mediator (Additional file 4).

### Feasibility

The average time needed to complete the questionnaire was 9 ± 5 minutes (range 5-39 minutes), well within our 45-minute target in all cases.

Participants rated three statements with the response options "agreed totally", "agreed partially", "disagreed partially" and "disagreed totally". To the first, "The length of the questionnaire is good", responses were 78.2%, 16.7%, 2.5% and 2.5% respectively. To the comprehensibility question, "I understood all questions well", the corresponding percentages were 80.8%, 17.6%, 1.2% and 0.4%. Finally, to the simplicity question, "It was easy to answer the questions", they were 77.9%, 20.0%, 1.3% and 0.7%.

Importantly, the percentages among participants below the age of 11 years were in the same ranges (length: 76.5%, 18.3%, 4.3% and 0.9%; comprehensibility: 73.7%, 22.8%, 3.5% and 0.0%; simplicity: 66.7%, 28.9%, 2.6% and 1.8%).

### Diagnostic accuracy

The sensitivity, specificity, PPV and NPV for diagnosing migraine and TTH according to the questionnaire are shown in Table 1. Compared to the first version of the questionnaire, sensitivities for migraine and TTH were higher in the second version; sensitivity was generally lower for TTH than for migraine.

**Table 1:** Diagnostic accuracy of the first and second versions of the Child/Adolescent HARDSHIP questionnaire for diagnosing migraine and tension-type headache according to ICHD-2

**Questionnaire, version 1.0**		**Sensitivity**	**Specificity**	**PPV**	**NPV**
**Migraine**		0.63	0.73	0.67	0.70
**TTH**		0.52	0.93	0.85	0.71
**Questionnaire, version 2.0**					
**Migraine**	All	0.71	0.76	0.76	0.71
	Children	0.75	0.63	0.71	0.68
	Adolescents	0.69	0.79	0.82	0.74
**TTH**	All	0.61	0.85	0.78	0.72
	Children	0.55	0.77	0.55	0.77
	Adolescents	0.63	0.93	0.91	0.68

Regarding general diagnostic agreement between questionnaire and interview, the kappa coefficient was 0.42 for the first version of the questionnaire applied in pupils aged 11-17 years. For the second version it was 0.47 overall, 0.40 in children and 0.49 in adolescents.

### Internal consistency

Cronbach’s Alpha was 0.80 for the first version of the questionnaire and 0.83 for the second version, indicating good internal consistency.

In the entire group of 1,202 subjects, 7 of the 10 questions related to burden of headache used in both versions of the questionnaire showed a statistically significant correlation with headache duration and 9 correlated with headache severity. Among the 12 questions about quality of life, eight were related to each of occurrence or not of headache in the last year, headache duration and headache severity.

### Burden of headache

The crude 1-year prevalence of headache in the whole sample was 89.3%, of migraine 39.3% and of TTH 37.9%. The prevalence of headache on ≥15 days/month was 4.5% and of probable MOH 0.8%; both these estimates were based on the second version of the questionnaire.

Among the 1,073 pupils with headache during the last year, 20.7% lost at least one day of school during the preceding 4 weeks, 19.4% left school early on at least one day, and 48.8% reported at least one day on which they had not been able to do other activities they had wanted to. The corresponding proportions of pupils increased with headache severity: they were 16.0%, 16.0% and 38.1% for mild headache (n = 570), 22.4%, 20.6% and 59.5% for moderate headache (n = 411) and 42.5%, 35.6% and 69.8% for severe headache (n = 88). All differences were statistically highly significant (p < 0.001). Among the 472 pupils with migraine, 23.1% lost at least one school day during the preceding 4 weeks, 21.8% left school early on at least one day and 59.9% lost at least one day of other activities. Among the 455 pupils with TTH, the corresponding percentages were 11.4%, 11.0% and 30.8%. The differences between migraine and TTH were also statistically highly significant (p < 0.001). In the small group of 22 pupils with headache on ≥15 days/month, the percentages were 13.6%, 0.0% and 63.6%. Because headache on ≥15 days/month could be assessed reliably only in the second study, we refrained from including it in statistical significance analyses.

**Figure 1:**
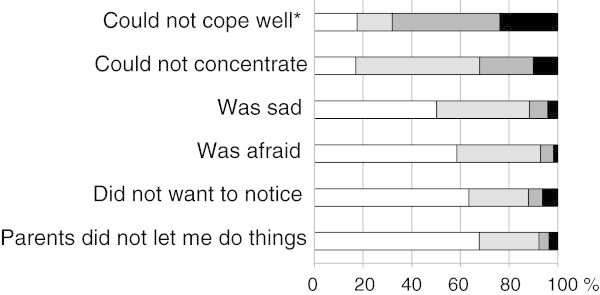
**Burden of headache in 1,073 pupils aged 6-17 years.** All statements refer to headache.White = never, light grey = sometimes, dark grey = often, black = always. *Wording of the statement and scores were reversed for the figure.

Their child’s headache caused parents of 7.1% of all pupils with headache in the last year to leave work on at least 1 day during the preceding 4 weeks. The proportions for mild, moderate and severe headache were 4.9%, 6.6% and 22.7% (p < 0.001) and those for migraine were 7.2% and TTH 4.4% (p = 0.07). Of pupils with headache on ≥15 days/month, 18.2% of parents had to leave work on at least 1 day during the preceding 4 weeks.The findings for the six questions on burden of headache, each rated on a 4-point Likert scale, are shown in Figure 1. The mean overall burden of headache summed score was 11.1 ± 2.8 (range 6-21). It increased with headache severity from 10.4 ± 2.5 for mild headache to 11.7 ± 2.8 for moderate headache and to 13.2 ± 2.8 for severe headache (p < 0.001). It was higher for migraine (11.7 ± 2.7) than for TTH (10.0 ± 2.4; p < 0.001), and higher for headache on ≥15 days/month (12.6 ± 2.9) than for migraine but not severe headache.

### Quality of life

**Figure 2:**
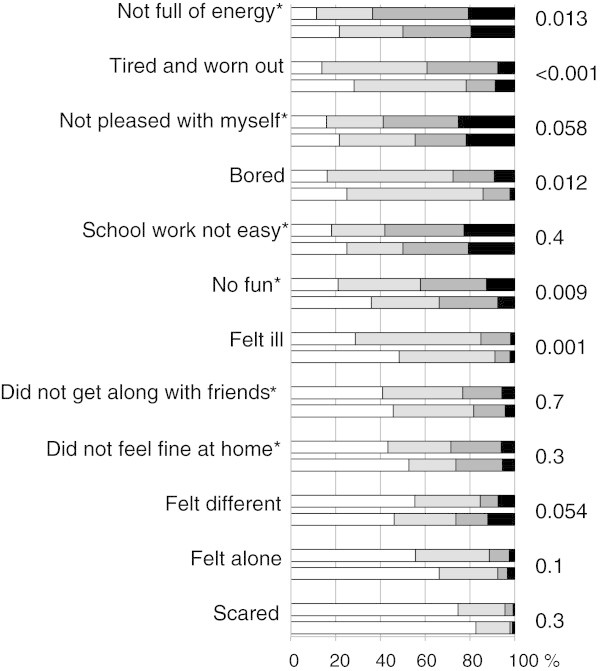
**Quality of life in 1,202 pupils aged 6-17 years.** White = never, light grey = sometimes, dark grey = often, black = always. For each item, the first row represents participants with headache during the last year, the second row those without. *Wording of the statement and scores were reversed for the figure.

The findings for the 12 questions on quality of life are shown in Figure 2. The mean quality of life summed score was 23.1 ± 4.9 in pupils without headache during the last year and 25.2 ± 5.1 in those with headache. It increased (*ie*, became worse) with headache severity from 24.4 ± 5.0 in mild headache to 25.7 ± 4.9 in moderate headache and to 27.4 ± 5.1 in severe headache (p < 0.001). It was higher in migraine (26.1 ± 4.8) than in TTH (23.6 ± 4.9; p < 0.001), and higher for headache on ≥15 days/month (27.9 ± 4.6) than migraine and severe headache.

### Headache yesterday

Using a new approach for estimating the burden of headache in children and adolescents, we analyzed the second version of the questionnaire completed by 491 pupils aged 6-17 years (mean 14.1 ± 3.3 years) with respect to headache occurring on the preceding day ("headache yesterday"). Headache yesterday was recorded by 29.6% of the pupils, whose headaches were associated with statistically significant differences from those reported by participants with headache in the last year but not yesterday: their headaches were more frequent, lasted longer and were more severe. In detail, the number of headache days during the previous 4 weeks was higher (7.8 ± 6.0 *vs* 2.7 ± 3.0; p < 0.001), headaches lasting ≥1 hour were more common (52.1% *vs* 36.0%; p < 0.001), and moderate or severe headache intensity was more usual (50.0% *vs* 34.0%, p = 0.004). The number of days of use of abortive headache medication during the preceding 4 weeks was higher in pupils with headache yesterday (2.6 ± 4.2 *vs* 1.0 ± 2.1 days; p < 0.001). Furthermore, a higher proportion of pupils with headache yesterday recorded at least one day of loss of own activities (64.3% *vs* 38.0%; p < 0.001) or parental work loss (11.1% *vs* 4.1%; p = 0.005). The burden of headache summed score (11.9 ± 2.6 *vs* 10.5 ± 2.8; p < 0.001) and quality of life score (27.2 ± 5.3 *vs* 24.8 ± 5.2; p < 0.001) were both significantly higher in pupils with headache yesterday than in others with headache last year.

### Comparison between Turkey and Austria

As this was the first study on burden of headache and quality of life in children and adolescents performed in two countries with an identical study design, we also compared the two and found several statistically significant differences.

Both the burden of headache summed score (11.4 ± 2.8 *vs* 10.7 ± 2.8; p < 0.001) and the quality of life summed score (25.6 ± 5.1 *vs* 24.5 ± 4.9; p < 0.001) were higher in Turkey than in Austria, indicating higher burden of headache and poorer quality of life with headache in Turkey. However, the latter finding must be set in the context of a higher quality of life summed score in Turkey than in Austria in pupils *without* headache (24.2 ± 4.8 vs 21.5 ± 4.7; p = 0.003), suggesting poorer quality of life in Turkey overall, and no greater effect in Turkey of headache upon it. This inference assumes the KINDL® questions on quality of life are culturally neutral.

Detailed analyses showed more differentiated findings. Days with school absenteeism, leaving school earlier, activity loss and parents’ work loss did not differ between the two countries. Burden of headache was higher in Turkey than in Austria with respect to being afraid of headache (p < 0.001), being restricted by parents because of headache (p = 0.037), being sad because of headache (p < 0.001), and not being able to cope well with headache (p < 0.001). In contrast, pupils in Austria reported more severe problems with concentrating because of headache (p < 0.001). Quality of life in pupils with headache during the last year was poorer in Turkey with respect to not having fun, feeling bored, feeling alone, not feeling fine at home and not doing schoolwork easily, whereas it was poorer in Austria with respect to feeling ill, feeling tired and worn out, not having energy and not being pleased with oneself (p < 0.001 for all).

## Discussion

This study on burden of headache in children and adolescents is unique for three reasons. First, it is the pilot for a global study involving countries from all regions of the world as defined by the World Health Organization. Second, it is the only study on the impact of headache in the young applying an identical design in two different countries. Third, this is the first study in children and adolescents using a new approach for estimating the burden of headache, namely headache yesterday.

### Key findings

These are the key findings. (a) The questionnaire developed for a global study in children and adolescents collected comprehensive information, was comprehensible to the participants, feasible and acceptable. (b) Prevalence and burden of headache were high. (c) Days lost because of headache increased with headache severity and more were lost from migraine than TTH. (d) The vast majority of pupils with headache experienced difficulties in coping with headache and in concentrating during headache. (e) Quality of life was poorer in pupils with headache than in those without. (f) Headache yesterday was a useful and confirmatory additional marker of the burden of headache. (g) Finally, burden of headache and quality of life differed somewhat between Turkey and Austria.

### Questionnaire

The second (final) versions of the Child and Adolescent HARDSHIP questionnaires included a total of 44 questions: one to record the date; two demographic questions; two screening questions for headache prevalence; 10 headache diagnostic questions (related to headache characteristics and associated symptoms); four questions enquiring into frequency of headache and of use of abortive medication (to capture MOH); four questions related to activity loss; three questions related to headache yesterday; six questions referring to other aspects of headache-attributed burden; 12 questions about quality of life. Only a small minority of pupils (1.6-5.0%, and 3.5-5.2% aged below 11 years) expressed any discontent with the length, comprehensibility or simplicity of the questionnaire. Thus, we succeeded in our aim of developing questionnaires that collect comprehensive information while being feasible for their purpose and acceptable to these age groups. To complete the information, we developed short additional questionnaires for the global study including questions about the school, its socio-economic background and details of the classes, and about numbers of participants and non-participants as well as reasons for non-participation.

The diagnostic accuracy of the questionnaire was acceptable, with better sensitivity (0.71) for migraine than for TTH (0.61). Even lower sensitivities for TTH have been found in studies in adults, which reflect the nature of TTH itself [9]. The kappa coefficient of 0.47 was within the range of studies in adults (0.39-0.59, with one outlier of 0.82). In the global study, ICHD-II criteria will be updated with those of ICHD-3 beta [18], but this will not have any significant impact.

Cronbach’s alpha of 0.83 indicated good internal consistency. The correlations of headache severity and duration to most of the burden of headache questions provide further evidence of internal consistency.

### Pilot studies

The pilot studies in Istanbul and Vienna ran successfully, with no significant practical problems. This is an important finding, indicating that the methodology of the study is also perfectly feasible for the proposed global study.

The participation rate was lower in Vienna than in Istanbul, a difference almost certainly explained by the additional requirement in Vienna for prior written parental consent rather than consent from the school authorities as in Istanbul. We believe the problem was passive non-response rather than active withholding of consent. In fact the rate in Istanbul (81.1%) was good, and the rate in Vienna (67.2%) not especially bad, when compared with adult studies from around the world [3,7]. For the global study, however, it will be highly desirable to keep the non-participation rate as low as possible, since selective participation is a clear source of potential bias [7]. We hope ethics committees will balance the societal value of the global study against its truly negligible potential for causing harm.

The findings of this pilot study will serve as the basis of sample size calculations, missing in almost all studies on the impact of headache in children and adolescents until now [5].

### Burden of headache

The 1-year prevalence of headache was high (89.3%) and of migraine very high (39.3%). Because this pilot study was not population-based, we make no more of the prevalence data.

Many days were lost because of headache. As might be expected, this impact increased with headache severity and was greater in migraine than in TTH. One fifth of pupils with headache reported losing at least one complete day from school within a period of 4 weeks; another fifth reported leaving school early at least once, implying the loss of part of a day. But almost half (48.8%) reported at least one day of lost time from other activities. It might appear that the effect on school time was less, but the questions were not phrased identically, reflecting the fact that it is far easier (and less consequential) to abandon or not undertake leisure activities than not go to school or leave early.

More than 80% of pupils felt at least sometimes that they were unable to cope well with headache and had difficulties in concentrating. These are not insignificant additional burdens at school.

A commonly overlooked but nonetheless quite important aspect of burden is impact on others. Of parents of pupils with headache, 7.1% had to leave *their* work on at least 1 day during the preceding 4 weeks. If this finding is replicated in the global study, it will uncover a very substantial but hitherto completely hidden component of headache burden with major societal implications.

### Quality of life

Quality of life was poorer in pupils with headache than in those without. Surprisingly, headache showed no statistically significant impact on school work, even though we expected this from clinical experience as well as from reports in the literature [19]. Analyzing the data separately for the two countries, we found a statistically highly significant impact of headache on school work in Austria, but not in Turkey. Pupils with and without headache alike reported more problems with schoolwork in Turkey than in Austria.

In the forthcoming global study, schools will not be selected by simple convenience sampling, but by modified cluster sampling technique which will reflect relevant diversities. Using this approach, and including a sufficient number of participants based on sample size calculations, we expect to eliminate a bias like this.

### Headache yesterday

Asking participants about headache yesterday offers the unique chance to (almost) eliminate recall bias. Currently, only two studies have been published applying this approach in adults [20,21]. In the present study, pupils with headache yesterday showed several differences from those with headache last year but not yesterday: headache frequency was higher, duration longer, intensity more severe and use of abortive headache medication more common. Pupils with headache yesterday recorded more days with loss of own activities and with parental work loss, and a higher overall burden of headache and poorer quality of life. To a large extent this reflects the fact that those responding positively to headache yesterday questions included most of those with headache on ≥15 days/month, who were the most heavily burdened. Enquiry into headache yesterday, therefore, not only is useful as an additional and confirmatory measure in epidemiological studies but also may offer effective screening for more severely affected headache patients in clinical practice.

### Comparison between Turkey and Austria

We found differences between the two countries with respect to burden of headache and quality of life in pupils with headache (as well as in headache-free pupils). These may or may not be socioculturally determined: these pilot studies were not population-based. As for the global study, it should be kept in mind that the methods developed for it are not intended to produce representative country samples, and the study will not be powered to produce estimates at country level. While global estimates are the principal objective, depending upon how many centres participate, and where these are, regional estimates should be possible alongside these.

### Limitations

We should discuss under this heading the decision to limit the retrospective enquiry to 4 weeks, although we will argue that it was sensible. It is supported by the study referred to earlier, comparing diary entries to retrospective recordings using PedMIDAS, which found recall accuracy was better at 30 than at 90 days [14]. The enquiry into lost days derives originally from the adult MIDAS [22], from which PedMIDAS itself evolved. For the individual patient in a therapeutic encounter, MIDAS counts days affected by headache in the previous 3 months as a compromise, balancing the need to reflect the illness over time in that individual against the increasing likelihood with time of recall error. In a population measure of burden using a large sample, there is no need to reflect the states of individuals, and therefore the questions can be applied, more reliably, to a shorter time of 4 weeks.

The limitation we must admit to arises from the definition in ICHD-II of subtypes of headache on ≥15 days/month: in particular, for a diagnosis of MOH, medication overuse must be present during >3 months [10]. This issue has been addressed elsewhere [4]. In cross-sectional population studies, all that can reasonably be ascertained is the concurrence in the last month of headache on ≥15 days and an excessive intake of acute drugs for headache (for a diagnosis of *probable* MOH). In practice, it has been argued, when frequent headache and medication overuse have coexisted for a month, both have usually been present for much longer [4]. This assertion may be more questionable in children.

## Conclusion

These pilot studies demonstrate the acceptability, validity and usefulness of the questionnaires, and feasibility and practicality of the proposed methodology, for a global assessment of the burden of headache in children and adolescents. Furthermore, they predict substantial impact of headache in these age groups.

The adult studies being undertaken by the Global Campaign against Headache [8] in many countries are completely rewriting our understanding of the burden of headache, showing that it has been very seriously underestimated worldwide [1,2]. In children and adolescents, with a much poorer knowledge base to begin with [3], we have little doubt our forthcoming global study will do the same. It is not before time.

## Competing interests

The authors declare that they have no competing interests.

## Authors’ contributions

ÇW-B initiated and conceived the study and contributed to data interpretation and drafting the manuscript. CW contributed to conception and design, analysis and interpretation of data, and drafted the manuscript. DU, UU, TS, MK, H-EZ and NTAG contributed to the design of the study, performed data acquisition and contributed to the interpretation of the data. GW contributed to the analysis and interpretation of data. AS contributed to conception and design of the study. TJS conceived the study, contributed to the interpretation of the data and revised the manuscript critically. All authors read and approved the final manuscript.

## Supplementary Material

Additional file 1 Child HARDSHIP questionnaire. Click here for file

Additional file 2 Adolescent HARDSHIP questionnaire. Click here for file

Additional file 3 Questionnaire for mediators and teachers, part 1. Click here for file

Additional file 4 Questionnaire for mediators and teachers, part 2. Click here for file
