# Supplementary material for: The global burden of headache in children and adolescents – developing a questionnaire and methodology for a global study
Source: J Headache Pain. 2014 Dec 11;15(1):86. doi: 10.1186/1129-2377-15-86 (PMC4273720; doi:10.1186/1129-2377-15-86)
Supplement: Additional file 2 — Adolescent HARDSHIP questionnaire. [file 1129-2377-15-86-S2.docx]

| ***Lifting The Burden***  Additional file 2  **in Official Relations with the World Health Organization**  **The Global Campaign against Headache**  **Adolescent HARDSHIP (Headache-attributed restriction, disability, social handicap and impaired participation) questionnaire**  **for mediated-group self-administration by adolescents aged 12-17 years** |
| --- |

| **Centre identifier**  (to be completed by the centre) | | | | | **__________** | | | | | | | |
| --- | --- | --- | --- | --- | --- | --- | --- | --- | --- | --- | --- | --- |
| **School identifier**  (to be completed by the centre) | | | | | **__________** | | | | | | | |
| **Class identifier**  (to be completed by the centre) | | | | | **__________** | | | | | | | |
| **Respondent identifier**  (to be completed by the interviewer) | | | | | **__________** | | | | | | | |
| **1** | | What is today’s date | | | **_____/_____/_______ day / month / year** | | | | | | | |
| **Questions about you** | | | | | | | | | | | | |
| **2** | How old are you? | | | | **_____ years** | | | | | | | |
| **3** | Are you male or female?  (please tick one box) | | | | **male** □ **female** □ | | | | | | | |
| **Screen questions** | | | | | | | | | | | | |
| **4** | Have you ever had a headache?  (please tick one box) | | | | **no** □ **yes** □ | | | | | | | |
| **5** | Have you had a headache in the last year?  (please tick one box) | | | | **no** □ **yes** □  If you answered no, please go straight to question 33. | | | | | | | |
| **Diagnostic questions** | | | | | | | | | | | | |
| These questions describe your headaches. | | | | | | | | | | | | |
| **6** | How long does your headache usually last?  (please tick one box) | | □  **less than 1 hour** | | □  **1-2 hours** | | □  **2-4 hours** | | | | □  **more than 4 hours** | |
| **7** | How bad is your headache usually?  (please tick one box) | | | | □  **not bad** | | □  **quite bad** | | | | □  **very bad** | |
| **8** | Which best describes your headache?  (please choose the one that is closest, and tick one box) | | | | □  **throbbing or pulsating (like the heart beat)** | | | | | □  **pressing** | | |
| **9** | Is your headache usually on only one side of the head, in the middle or on both sides?  (please tick one box) | | | | □  **on one side** | | □  **in the middle** | | | | □  **on both sides** | |
| **10** | Does exercise (like walking or climbing stairs) make your headache worse?  (please tick one box) | | | | **no** □ **yes** □ | | | | | | | |
| **11** | Do you avoid exercise (like walking or climbing stairs) when you have a headache?  (please tick one box) | | | | **no** □ **yes** □ | | | | | | | |
| **12** | With your headache, do you usually feel sick (as though you may throw up)?  (please tick one box) | | | | **no** □ **yes** □ | | | | | | | |
| **13** | With your headache, are you usually actually sick (do you throw up)?  (please tick one box) | | | | **no** □ **yes** □ | | | | | | | |
| **14** | When you have a headache, do you prefer to be in the dark?  (please tick one box) | | | | **no** □ **yes** □ | | | | | | | |
| **15** | When you have a headache, do you prefer to be in the quiet?  (please tick one box) | | | | **no** □ **yes** □ | | | | | | | |
| **Impact questions** | | | | | | | | | | | | |
| The questions from now on are about how your headaches affect you and your life.  The first two questions are about **last week**. | | | | | | | | | | | | |
| **16** | On how many days **in the last week** did you have a headache?  (please enter the number of days, between 0 and 7) | | | | | | | | **_____ day(s)** | | | |
| **17** | On how many days **in the last week** did you take medicine or pills because of headache?  (please enter the number of days, between 0 and 7) | | | | | | | | **_____ day(s)** | | | |
| The next questions are about the **last four weeks**. | | | | | | | | | | | | |
| **18** | On how many days **in the last four weeks** did you have a headache?  (please enter the number of days, between 0 and 28) | | | | | | | | **_____ day(s)** | | | |
| **19** | On how many days **in the last four weeks** did you take medicine or pills because of headache?  (please enter the number of days, between 0 and 28) | | | | | | | | **_____ day(s)** | | | |
| **20** | On how many days **in the last four weeks** did you not go to school because of your headaches?  (please enter the number of days, between 0 and 20) | | | | | | | | **_____ day(s)** | | | |
| **21** | On how many days **in the last four weeks** did you leave school early because of your headaches?  (please enter the number of days, between 0 and 20) | | | | | | | | **_____ day(s)** | | | |
| **22** | On how many **days in the last four weeks** could you not do things you wanted to because of your headaches?  (please enter the number of days, between 0 and 28) | | | | | | | | **_____ day(s)** | | | |
| **23** | During the **last four weeks**, have **your** headaches caused your parents to lose time from work?  (please tick one box and, if yes, enter the total number of days lost) | | | | **no** □ **yes** □  **_____ day(s)** | | | | | | | |
| **Yesterday questions** | | | | | | | | | | | | |
| **24** | Did you have a headache **yesterday**?  (please tick one box) | | | | **no** □ **yes** □  If you answered no, please go straight to question 27. | | | | | | | |
| **25** | How bad was it?  (please tick one box) | | | | □  **not bad** | | □  **quite bad** | | | | □  **very bad** | |
| **26** | Did you miss school yesterday?  (please tick one box) | | | | □  **no** | | □  **yes, I left early** | | | | □  **yes, I did not go** | |
| **More impact questions** | | | | | | | | | | | | |
| Please think about **the last four weeks** to answer these questions. | | | | | | | | | | | | |
| **27** | I was afraid of having a headache  (please tick one box) | | | □  **never** | | □  **sometimes** | | □  **often** | | | | □  **always** |
| **28** | My parents did not let me do things because of my headaches  (please tick one box) | | | □  **never** | | □  **sometimes** | | □  **often** | | | | □  **always** |
| **29** | I could not concentrate because of my headaches  (please tick one box) | | | □  **never** | | □  **sometimes** | | □  **often** | | | | □  **always** |
| **30** | I was sad because of my headaches  (please tick one box) | | | □  **never** | | □  **sometimes** | | □  **often** | | | | □  **always** |
| **31** | I was able to cope well with my headaches  (please tick one box) | | | □  **never** | | □  **sometimes** | | □  **often** | | | | □  **always** |
| **32** | I wanted nobody to notice my headaches  (please tick one box) | | | □  **never** | | □  **sometimes** | | □  **often** | | | | □  **always** |
| The remaining questions are for **everyone**, with or without headaches. | | | | | | | | | | | | |
| **Quality of life questions** | | | | | | | | | | | | |
| Please think about **your life in the last four weeks** to answer these questions. | | | | | | | | | | | | |
| **33** | I felt ill  (please tick one box) | | | □  **never** | | □  **sometimes** | | □  **often** | | | | □  **always** |
| **34** | I was tired and worn-out  (please tick one box) | | | □  **never** | | □  **sometimes** | | □  **often** | | | | □  **always** |
| **35** | I felt full of energy  (please tick one box) | | | □  **never** | | □  **sometimes** | | □  **often** | | | | □  **always** |
| **36** | I had fun and laughed a lot  (please tick one box) | | | □  **never** | | □  **sometimes** | | □  **often** | | | | □  **always** |
| **37** | I was bored  (please tick one box) | | | □  **never** | | □  **sometimes** | | □  **often** | | | | □  **always** |
| **38** | I felt alone  (please tick one box) | | | □  **never** | | □  **sometimes** | | □  **often** | | | | □  **always** |
| **39** | I was scared  (please tick one box) | | | □  **never** | | □  **sometimes** | | □  **often** | | | | □  **always** |
| **40** | I felt pleased with myself  (please tick one box) | | | □  **never** | | □  **sometimes** | | □  **often** | | | | □  **always** |
| **41** | I felt fine at home  (please tick one box) | | | □  **never** | | □  **sometimes** | | □  **often** | | | | □  **always** |
| **42** | I got along with my friends  (please tick one box) | | | □  **never** | | □  **sometimes** | | □  **often** | | | | □  **always** |
| **43** | I felt different from other children  (please tick one box) | | | □  **never** | | □  **sometimes** | | □  **often** | | | | □  **always** |
| **44** | Doing my schoolwork was easy  (please tick one box) | | | □  **never** | | □  **sometimes** | | □  **often** | | | | □  **always** |
| **This is the end of the questionnaire. Thank you very much for answering it.** | | | | | | | | | | | | |
