# Supplementary material for: The global burden of headache in children and adolescents – developing a questionnaire and methodology for a global study
Source: J Headache Pain. 2014 Dec 11;15(1):86. doi: 10.1186/1129-2377-15-86 (PMC4273720; doi:10.1186/1129-2377-15-86)
Supplement: Additional file 3 — Questionnaire for mediators and teachers, part 1. [file 1129-2377-15-86-S3.docx]

***Lifting The Burden***

Additional file 3

**in Official Relations with
the World Health Organization**

**The Global Campaign against Headache**

**Research Project:
The global burden attributable to childhood and adolescent headache disorders**

(children aged 6-11 years and adolescents aged 12-17 years)

**Questionnaire for mediators and teachers (part 1)**

**(version 2.1)**

**Page 1 to be completed centrally**

| **Country** | **_____________** |
| --- | --- |
| Income according to World Bank (check one box only) | □ high □ upper middle □ lower middle □ low |
| Consents required (check all that apply) | □ ethics committee □ pupil (mandatory) □ parents □ teacher □ school manager □ other school authorities □ local political authorities □ State (national) political authorities |

| **These questions to be answered by school (teacher and/or mediator)** | |
| --- | --- |
| **School identifier** | ____________________________ |
| **School details** |  |
| Location of school (check one box only) | □ urban  □ semi-urban or mixed urban/rural  □ rural |
| Total numbers of pupils in the school | aged 6-11 years ________  aged 12-17 years ________ |
| What proportion of pupils are estimated to come from low-income families? (check one box only) | □ less than a quarter  □ between a quarter and a half  □ between half and three quarters  □ more than three quarters |
| What proportion of pupils must travel for more than 1 hour to and from school (each way)? (check one box only) | □ less than a quarter  □ between a quarter and a half  □ between half and three quarters  □ more than three quarters |
| Number of classes at this school in each age range  (two or more age categories may be bracketed together if appropriate) | aged 6-7 years ________  aged 7-8 years ________  aged 8-9 years ________  aged 9-10 years ________  aged 10-11 years ________  aged 11-12 years ________  aged 12-13 years ________  aged 13-14 years________  aged 14-15 years ________  aged 15-16 years ________  aged 16-17 years ________ |
