# Supplementary material for: The global burden of headache in children and adolescents – developing a questionnaire and methodology for a global study
Source: J Headache Pain. 2014 Dec 11;15(1):86. doi: 10.1186/1129-2377-15-86 (PMC4273720; doi:10.1186/1129-2377-15-86)
Supplement: Additional file 4 — Questionnaire for mediators and teachers, part 2. [file 1129-2377-15-86-S4.docx]

***Lifting The Burden***

**in Official Relations with
the World Health Organization**

**The Global Campaign against Headache**

**Research Project:
The global burden attributable to childhood and adolescent headache disorders**

Additional file 4

(children aged 6-11 years and adolescents aged 12-17 years)

**Questionnaire for mediators and teachers (part 2)**

**(version 2.1)**

| **Centre identifier** |  |
| --- | --- |
| **School identifier** |  |
| **These questions to be answered by school (teacher and/or mediator)** | |
| **General class details** |  |
| Number of classes invited to participate | _____ |
| Did any classes decline to participate? (check one box) | □ no □ yes  If yes, please write below which ones (age range) and why |
|  | |
| **Details of each participating class** (to be completed separately for each class) | |
| **Class 1** | |
| Age range | ________________ years |
| Total numbers of pupils in this class | boys _______ girls _______ |
| Numbers of pupils asked to participate | boys _______ girls _______ |
| Numbers of pupils not participating | boys _______ girls _______ |
| Reasons for not participating (give numbers only) | pupil withheld consent _____  parent(s) withheld consent _____  pupil not present _____ |
| **Class 2** | |
| Age range | ________________ years |
| Total numbers of pupils in this class | boys _______ girls _______ |
| Numbers of pupils asked to participate | boys _______ girls _______ |
| Numbers of pupils not participating | boys _______ girls _______ |
| Reasons for not participating (give numbers only) | pupil withheld consent _____  parent(s) withheld consent _____  pupil not present _____ |
| **Class 3** | |
| Age range | ________________ years |
| Total numbers of pupils in this class | boys _______ girls _______ |
| Numbers of pupils asked to participate | boys _______ girls _______ |
| Numbers of pupils not participating | boys _______ girls _______ |
| Reasons for not participating (give numbers only) | pupil withheld consent _____  parent(s) withheld consent _____  pupil not present _____ |
| **Class 4** | |
| Age range | ________________ years |
| Total numbers of pupils in this class | boys _______ girls _______ |
| Numbers of pupils asked to participate | boys _______ girls _______ |
| Numbers of pupils not participating | boys _______ girls _______ |
| Reasons for not participating (give numbers only) | pupil withheld consent _____  parent(s) withheld consent _____  pupil not present _____ |
| **Class 5** | |
| Age range | ________________ years |
| Total numbers of pupils in this class | boys _______ girls _______ |
| Numbers of pupils asked to participate | boys _______ girls _______ |
| Numbers of pupils not participating | boys _______ girls _______ |
| Reasons for not participating (give numbers only) | pupil withheld consent _____  parent(s) withheld consent _____  pupil not present _____ |
| **Class 6** | |
| Age range | ________________ years |
| Total numbers of pupils in this class | boys _______ girls _______ |
| Numbers of pupils asked to participate | boys _______ girls _______ |
| Numbers of pupils not participating | boys _______ girls _______ |
| Reasons for not participating (give numbers only) | pupil withheld consent _____  parent(s) withheld consent _____  pupil not present _____ |
| **If there are more than 6 classes, use add-on sheets** | |
| **Add-on sheet** | |
| **Class ____** | |
| Age range | ________________ years |
| Total numbers of pupils in this class | boys _______ girls _______ |
| Numbers of pupils asked to participate | boys _______ girls _______ |
| Numbers of pupils not participating | boys _______ girls _______ |
| Reasons for not participating (give numbers only) | pupil withheld consent _____  parent(s) withheld consent _____  pupil not present _____ |
| **Class ____** | |
| Age range | ________________ years |
| Total numbers of pupils in this class | boys _______ girls _______ |
| Numbers of pupils asked to participate | boys _______ girls _______ |
| Numbers of pupils not participating | boys _______ girls _______ |
| Reasons for not participating (give numbers only) | pupil withheld consent _____  parent(s) withheld consent _____  pupil not present _____ |
| **Class ____** | |
| Age range | ________________ years |
| Total numbers of pupils in this class | boys _______ girls _______ |
| Numbers of pupils asked to participate | boys _______ girls _______ |
| Numbers of pupils not participating | boys _______ girls _______ |
| Reasons for not participating (give numbers only) | pupil withheld consent _____  parent(s) withheld consent _____  pupil not present _____ |
